# On the conspecificity of *Carinaulus coreensis* (Kim) and *C. inexpectatus* (Balthasar) (Coleoptera, Scarabaeidae, Aphodiinae)

**DOI:** 10.3897/BDJ.13.e169855

**Published:** 2025-11-05

**Authors:** Jaeil Shim, Jeong-Hun Song

**Affiliations:** 1 Department of Agricultural Biology, National Institute of Agricultural Sciences, Wanju, Republic of Korea Department of Agricultural Biology, National Institute of Agricultural Sciences Wanju Republic of Korea; 2 Department of Biology, Chungnam National University, Daejeon, Republic of Korea Department of Biology, Chungnam National University Daejeon Republic of Korea

**Keywords:** Aphodiinae, *
Carinaulus
*, *Carinaulus coreensis*, *Carinaulus inexpectatus*, dung beetle, synonym

## Abstract

**Background:**

The genus *Carinaulus* Tesař, belonging to the scarab beetle subfamily Aphodiinae, currently includes 20 valid species distributed across the Eastern Palearctic and Oriental Regions. The genus is characterised by the following elytral features: broad and deep striae; striae prominently punctate and distinctly crenulate; intervals tectiform with a median carina; and the sixth and eighth intervals joined at the pre-apical area, though lacking a carina in that region. Two species are recognised in the Korean Peninsula, *Carinaulus inexpectatus* (Balthasar, 1935) in North Korea and *Carinaulus coreensis* (Kim, 1986) in South Korea. *Carinaulus inexpectatus* (Balthasar, 1935), with Vladivostok designated as its type locality, occurs in the Russian Far East and North Korea, whereas *C. coreensis* (Kim, 1986) is considered endemic to South Korea.

**New information:**

During examination of Korean *Carinaulus* Tesař, we compared several specimens of *Carinaulus coreensis* (Kim, 1986), including a paratype and *C. inexpectatus* (Balthasar, 1935) collected from North Korea. We found that the two nominal species cannot be distinguised by the morphological characters, including labrum chaetotaxy and male genitalia. Accordingly, it is proposed that *Aphodius coreensis* Kim, 1986, is a subjective junior synonym of *Aphodius nexpectatus* Balthasar, 1935.

## Introduction

The genus *Carinaulus* Tesař, 1945 erected for *Aphodius vseteckai* Tesař, 1945 from China (Tesař 1945), belonging to the subfamily Aphodiinae of the family Scarabaeidae, currently includes 20 species. These dung beetle species are largely distributed in the Palaearctic Region, with only a single species occurring in the Oriental Region (Dellacasa et al. 2016, Dellacasa and Dellacasa 2006, Schoolmeesters 2025). The morphological features of *Carinaulus* have been studied extensively (Červenka 2000, Dellacasa et al. 2001, Král et al. 2021, Minkina 2022).

Two nominal species of *Carinaulus* have been reported on the Korean Peninsula. The first, *C. inexpectatus* (Balthasar, 1935), has its type locality at Vladivostok in the Russian Far East and has been recorded in North Korea (Stebnicka 1980). The second, *C. coreensis* (Kim, 1986), has been regarded as an endemic species confined to South Korea.

During a taxonomic review of Korean *Carinaulus*, we examined the type material of *C. coreensis* together with North Korean specimens of *C. inexpectatus*. Detailed comparisons of external morphology, including the male genitalia and epipharynx, revealed intraspecific variation in several characters (epipharynx: length and number of celtes; meso- and metatibiae: length of marginal spinules), with no consistent interspecific differences. We therefore conclude that the two are conspecific and we synonymise *Carinaulus coreensis* (Kim, 1986) with *Carinaulus inexpectatus* (Balthasar, 1935). A re-description, diagnostic characters and habitus photographs of *C. inexpectatus* are presented herein.

## Materials and methods

Two paratype females and one additional female of *C. coreensis* (Kim, 1986), together with three males of *C. inexpectatus* (Balthasar, 1935) from Mt. Kumgang, North Korea are deposited in the National Institute of Biological Resources Insect Collection (NIBR, Incheon, Korea). In addition, two specimens of *C. coreensis* from Gangwon-do, South Korea are housed in the National Agricultural Science Insect Collection (NASIC, Wanju, Korea). A high-resolution photograph of the holotype of *C. inexpectatus* was supplied by the Czech Natural History Museum Insect Collection (CHM, Prague, Czechia) for comparative analyses.

Specimens were examined under an Olympus SZX-16 stereomicroscope fitted with an Olympus DP-71 digital camera (Olympus Corporation, Tokyo, Japan). Habitus and structural images were obtained using a Canon EOS 5D DSLR camera, equipped with a Canon MP-E 65 mm f/2.8 1–5× macro lens. Focus stacking and merging were performed using Helicon Focus 5.3 (Helicon Soft Ltd., Kharkiv, Ukraine) and images were post-processed using Adobe Photoshop CC 2020 (Adobe Systems, San Jose, CA, USA).

To remove soft tissue of the male genitalia (aedeagi) and epipharynx, specimens were immersed in a 10% sodium hydroxide solution at 60°C for 3–4 hours.

Morphological terminology follows Dellacasa et al. (2001) and epipharynx terminology follows Dellacasa et al. (2010).

## Taxon treatments

### Carinaulus

Tesař, 1945

8F7DEFEE-079A-568E-987D-7EBB3AFC0C29

https://www.checklistbank.org/dataset/1027/taxon/18-.10-.01-.01-.036-.000-.000-.-

Carinaulus (Tesař, 1945) Tesař (1945): 66. Type species: *Aphodius vseteckai* Tesař, 1945 = *Carinaulus vseteckai* (Tesař, 1945). Elevated to genus by Dellacasa et al. (2001): 113.
Aphodius (Carinaulus) Tesař, 1945 Tesař (1945): 66. Type species: *Aphodius vseteckai* Tesař, 1945 = *Carinaulus vseteckai* (Tesař, 1945).
Aphodius (Oxyaphodius) Balthasar, 1965 Balthasar (1965): 314. Type species: Aphodius (Oxyaphodius) sikkimensis Balthasar, 1965 = *Carinaulus sikkimensis* (Balthasar, 1965). syn. by Červenka (2000): 30.
Aphodius (Carinaulus) vseteckai Tesař, 1945

#### Diagnosis

The members of the genus *Carinaulus* can be distinguished from other Aphodiinae taxa by the following combination of characteristics [modified from Červenka (2000) and Dellacasa et al. (2001)]: clypeus wide, margin weakly to moderately concave at the middle; with or without cephalic tubercles (occasionally in sexual diamorphism); elytral striae wide and deep, strial puncture distinctly crenulated; intervals tectiform or carinate; sixth and eighth intervals joined before pre-apical area; carina generally lacking in apical area of elytral intervals; meso- and metatibia apical spinules more or less equal in length, exceptionally unequal in length.

### Carinaulus inexpectatus

(Balthasar, 1935)

4A69FA2E-F665-5F15-8DAE-8ADCCD38E642

https://www.checklistbank.org/dataset/1027/taxon/18-.10-.01-.01-.036-.000-.009-.-

Aphodius (Agrilinus) inexspectatus Balthasar, 1935 [original spelling] Balthasar (1935): 121. — spelling later corrected to *inexpectatus* by subsequent authors. comb. in *Carinaulus* by Červenka (2000): 44.
Aphodius (Carinaulus) nigrocarinatus Nikolajev, 1979 Nikolajev (1979): 99. syn. by Stebnicka and Galante (1991): 727.
Aphodius (Carinaulus) coreensis Kim, 1986 Kim (1986): 8. **syn. nov.**
**Records from the Korean Peninsula**
Agrilinus inexpectatus : Minkina (2022): 237 (North Korea).
Aphodius (Agrilinus) inexpectatus : Stebnicka (1980): 243 (North Korea); Stebnicka and Galante (1991): 727 (North Korea).
Aphodius (Agrilinus) inexspectatus : Kim (1989): 4 (North Korea); Kim (2000): 100 (North Korea); ESK and KSAE (1994): 148 (North Korea) [Korean Checklist].
Aphodius (Carinaulus) coreensis : Kim (1986): 8 (South Korea); Kim (2000): 87 (South Korea); Dellacasa and Dellacasa (2006): 117 (North and South Korea) [Palaearctic Catalogue]; Kim (2012): 108 (South Korea); KSAE and ESK (2021): 262 (South Korea) [Korean Checklist].
Aphodius (Carinaulus) inexspectatus : Dellacasa and Dellacasa (2006): 117 (North Korea) [Palaearctic Catalogue]; Kim (2012): 109 (North Korea); KSAE and ESK (2021): 262 (North Korea) [Korean Checklist].
Carinaulus coreensis : Dellacasa et al. (2016): 116 (South Korea) [Palaearctic Catalogue].
Carinaulus inexpectatus : Dellacasa et al. (2016): 116 (North and South Korea) [Palaearctic Catalogue].

#### Materials

- **Type status:** Holotype. **Occurrence:** recordedBy: Hermann Frieb.; individualCount: 1; lifeStage: Adult; preparations: Dried specimen, photographed; disposition: in collection; occurrenceID: 3DFA0D2C-E8BD-5721-B54B-02519BB5F1DD; **Taxon:** scientificName: *Carinaulus inexpectatus* (Balthasar, 1935); originalNameUsage: Aphodius (Agrilinus) inexpectatus Balthasar, 1935; namePublishedIn: Balthasar V (1935) Einige neue palaearktische Scarabaeidae. 27. Beitrag zur Kenntnis der *Scarabaeidae* des palaearktischen Faunengebietes. *Entomologische Blätter. Krefeld* 31(3):120–124.; kingdom: Animalia; phylum: Arthropoda; class: Insecta; order: Coleoptera; family: Scarabaeidae; genus: Carinaulus; specificEpithet: inexpectatus; taxonRank: species; scientificNameAuthorship: (Balthasar, 1935); nomenclaturalCode: ICZN; **Location:** higherGeography: Eurasia; country: Russia; countryCode: RU; stateProvince: Primorsky Krai; county: Vladivostok; verbatimLocality: Wladiwostok, Ost-Asien; **Identification:** identifiedBy: Ł. Minkina; dateIdentified: 2021-06; **Event:** eventDate: 19-VIII-1929; year: 1929; month: August; day: 19; **Record Level:** type: Dried specimen (photographed); institutionCode: CHM, Czech Natural History Museum, Prague, Czech Republic
- **Type status:** Paratype. **Occurrence:** recordedBy: J.I. Kim; individualCount: 2; sex: 2 females; lifeStage: Adults; preparations: dried specimen; disposition: In collection; occurrenceID: 54A073D1-B3C1-57E6-BD22-542F7F08254C; **Taxon:** scientificName: *Carinaulus coreensis* Kim, 1986; acceptedNameUsage: *Carinaulus coreensis* (Kim, 1986); originalNameUsage: Aphodius (Carinaulus) coreensis (Kim, 1986); namePublishedIn: Kim JI (1986) Taxonomic study on the Korean Laparosticti. V. Two new and two rare unrecorded species of the genus *Aphodius*. The Korean Journal of Entomology 16(1):7-11.; kingdom: Animalia; phylum: Arthropoda; class: Insecta; order: Coleoptera; family: Scarabaeidae; genus: Carinaulus; specificEpithet: coreensis; scientificNameAuthorship: (Kim, 1986); nomenclaturalCode: ICZN; **Location:** higherGeography: Asia; country: South Korea; countryCode: KR; stateProvince: Gangwon-do; county: Chuncheon-si; municipality: Namsan-myeon; locality: Seocheon-ri; verbatimLocality: Seucheun-ri, Nam-myun, Choonseung-kun, Kangwon-do; **Identification:** identifiedBy: J.I. Kim; identificationReferences: Kim JI (1986) Taxonomic study on the Korean Laparosticti. V. Two new and two rare unrecorded species of the genus Aphodius. The Korean Journal of Entomology 16(1):7-11.; **Event:** samplingProtocol: Cow dung; eventDate: 18. Ⅸ. 1983; year: 1983; month: September; day: 18; habitat: Cow dung; **Record Level:** type: Dried specimen; language: en; institutionCode: NIBR, ational Institute of Biological Resources, Incheon, Korea
- **Type status:** Other material. **Occurrence:** recordedBy: H. Steinmann & T. Vasarhelyi; individualCount: 3; sex: 3 males; lifeStage: Adults; preparations: dried specimen; disposition: In collection; occurrenceID: C849E26A-64D2-547D-85AA-D3ABB24B8B64; **Taxon:** scientificName: *Carinaulus inexpectatus* (Balthasar, 1935); originalNameUsage: Aphodius (Agrilinus) inexpectatus Balthasar, 1935; namePublishedIn: Balthasar V (1935) Einige neue palaearktische Scarabaeidae. 27. Beitrag zur Kenntnis der *Scarabaeidae* des palaearktischen Faunengebietes. *Entomologische Blätter. Krefeld* 31(3):120–124.; kingdom: Animalia; phylum: Arthropoda; class: Insecta; order: Coleoptera; family: Scarabaeidae; genus: Carinaulus; specificEpithet: inexpectatus; scientificNameAuthorship: (Balthasar, 1935); nomenclaturalCode: ICZN; **Location:** higherGeography: Asia; country: North Korea; countryCode: KP; stateProvince: Gangwon-do; county: Geumgang-gun; locality: Mt. Geumgang; verbatimLocality: North Korea, Mt. Geumgang; **Identification:** identifiedBy: J. Shim; **Event:** eventDate: 28. Sep. 1979; year: 1979; month: September; day: 28; verbatimEventDate: 28. Sep. 1979; **Record Level:** type: Dried specimen; language: en; institutionCode: NIBR, ational Institute of Biological Resources, Incheon, Korea
- **Type status:** Other material. **Occurrence:** recordedBy: NIAST, National Institute of Agricultural Sciences.; individualCount: 1; sex: 1 female; lifeStage: Adult; preparations: dried specimen; disposition: In collection; occurrenceID: A0A58553-30F1-54DF-9791-B835B4683B4B; **Taxon:** scientificName: *Carinaulus coreensis* (Kim, 1986); originalNameUsage: Aphodius (Carinaulus) coreensis Kim, 1986; namePublishedIn: Kim JI (1986) Taxonomic study on the Korean Laparosticti. V. Two new and two rare unrecorded species of the genus *Aphodius*. The Korean Journal of Entomology 16(1):7-11.; kingdom: Animalia; phylum: Arthropoda; class: Insecta; order: Coleoptera; family: Scarabaeidae; genus: Carinaulus; specificEpithet: coreensis; scientificNameAuthorship: (Kim, 1986); nomenclaturalCode: ICZN; **Location:** higherGeography: Asia; country: South Korea; countryCode: KR; stateProvince: Gangwon-do; county: Pyeongchang-gun; verbatimLocality: Gangwon-do, Pyeongchang-gun; **Identification:** identifiedBy: J.I. Kim; identificationReferences: Kim JI (1986) Taxonomic study on the Korean Laparosticti. V. Two new and two rare unrecorded species of the genus Aphodius. The Korean Journal of Entomology 16(1):7-11.; **Event:** samplingProtocol: Cow dung; eventDate: 2. Sep. 1997; year: 1997; month: September; day: 2; verbatimEventDate: 2 Sep 1997; habitat: Cow dung; **Record Level:** type: Dried specimen; language: en; institutionCode: NIBR, ational Institute of Biological Resources, Incheon, Korea
- **Type status:** Other material. **Occurrence:** recordedBy: T.W. Kang; individualCount: 2; sex: 1 male, 1 female; lifeStage: Adults; preparations: dried specimen, pinned; disposition: In collection; occurrenceID: EB881714-FD20-5671-9E33-F86B7D4186F2; **Taxon:** scientificName: *Carinaulus coreensis* (Kim, 1986); originalNameUsage: Aphodius (Carinaulus) coreensis Kim, 1986; namePublishedIn: Kim JI (1986) Taxonomic study on the Korean Laparosticti. V. Two new and two rare unrecorded species of the genus *Aphodius*. The Korean Journal of Entomology 16(1):7-11.; kingdom: Animalia; phylum: Arthropoda; class: Insecta; order: Coleoptera; family: Scarabaeidae; genus: Carinaulus; specificEpithet: coreensis; scientificNameAuthorship: (Kim, 1986); nomenclaturalCode: ICZN; **Location:** higherGeography: Asia; country: South Korea; countryCode: KR; stateProvince: Gangwon-do; county: Inje-gun; municipality: Sangnam-myeon; verbatimLocality: Sangnam-myeon, Inje-gun; **Identification:** identifiedBy: J.I. Kim & T.W. Kang; dateIdentified: 28. Ⅸ. 2000; identificationReferences: Kim JI (1986) Taxonomic study on the Korean Laparosticti. V. Two new and two rare unrecorded species of the genus Aphodius. The Korean Journal of Entomology 16(1):7-11.; **Event:** samplingProtocol: Cow dung; eventDate: 28. Ⅸ. 2000; year: 2000; month: September; day: 28; verbatimEventDate: 28 Ⅸ 2000; **Record Level:** type: Dried specimen; language: en; institutionCode: NASIC, Department of Agricultural Biology, National Institute of Agricultural Sciences, Wanju, Korea

#### Re-description

**Total body length**: 5.1−6.7 mm. Medium to large-sized beetles.

**Figure 1.**
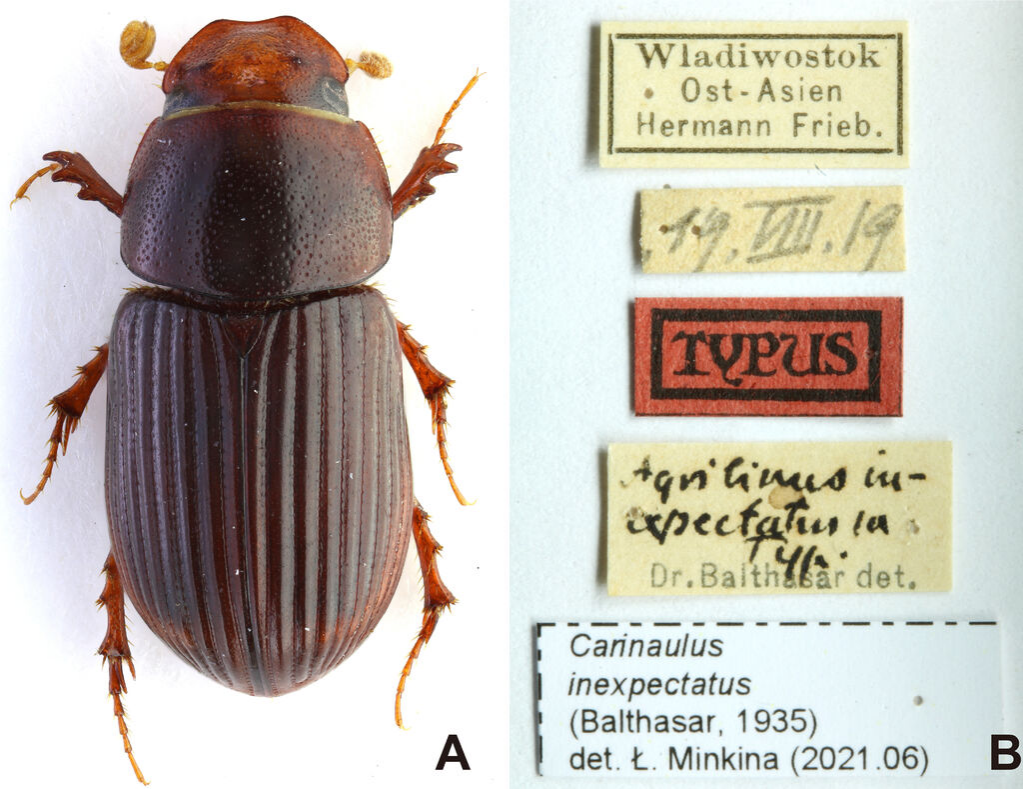
Holotype specimen of *Carinaulus inexpectatus* (Balthasar, 1935). **A** habitus, dorsal aspect, 5.5 mm; **B** holotype label.

**Figure 2.**
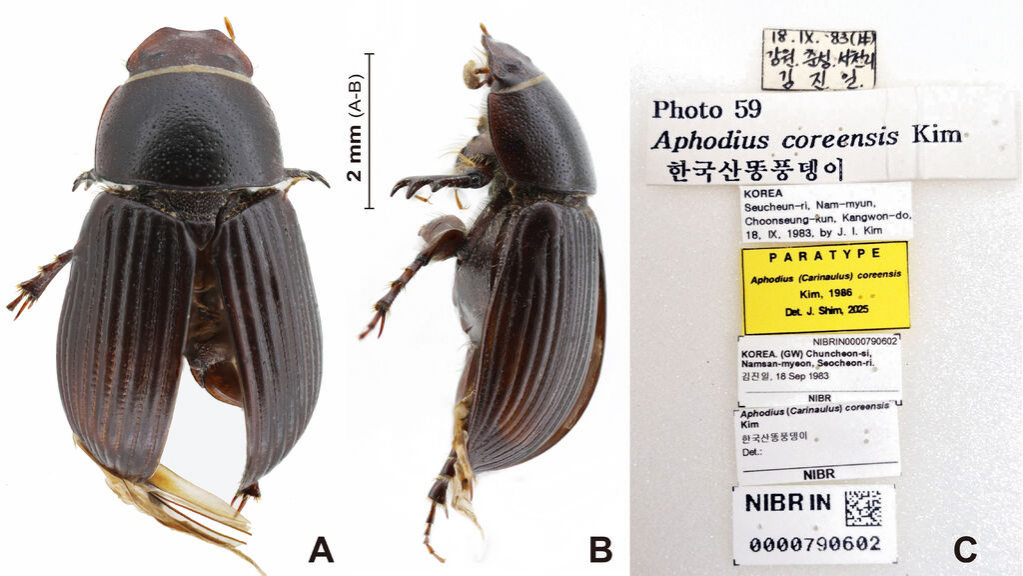
Paratype specimen of *Carinaulus coreensis* (Kim, 1986) syn. nov., abdomen dissected. **A** habitus, dorsal aspect; **B** habitus, lateral aspect; **C** paratype label.

**Figure 3.**
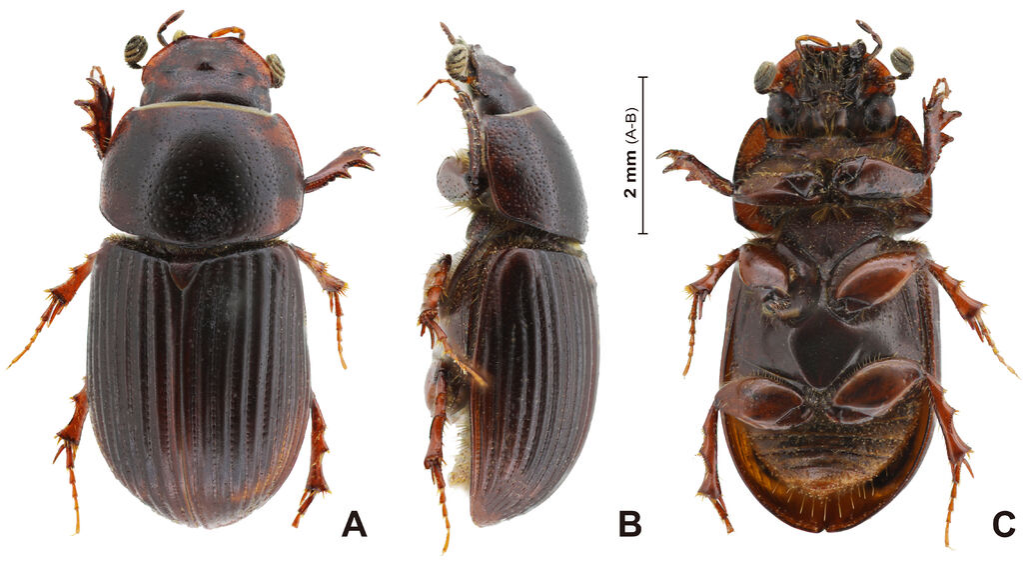
*Carinaulus inexpectatus*, male habitus. **A** dorsal aspect; **B** lateral aspect; **C** ventral aspect.

**Colouration** (Fig. 1A; Fig. 2A; Fig. 3A): Body colour reddish-brown to brown, surface shiny. Antenna lamellate colour yellowish.

**Male** (Fig. 3A): Body elongate oval shape.

**Figure 4.**
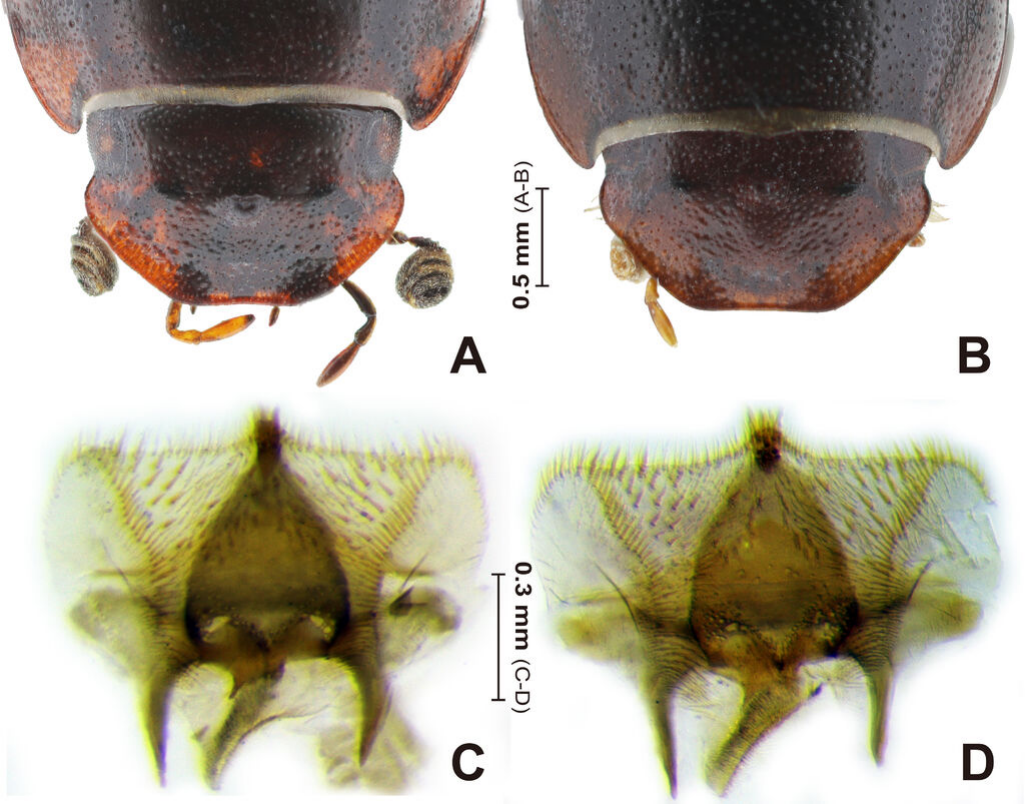
*Carinaulus inexpectatus*, head and epipharynx. **A** male head; **B** female head; **C–D** epipharynx: *C. coreensis* **syn. nov.** (**C**); *C. inexpectatus* (**D**).

**Head** (Fig. 4A): Clypeal margin rim distinct and thin, somewhat distinctly reflexed; clypeus moderately bordered, anterior margin weakly concave at the middle, its lateral apex area widely arcuate, antero-lateral margin nearly transverse. Genae rather strongly protruded laterally, lateral margin arcuate, slightly exceeding eyes; dorsal surface punctation structure similar with frontal ones; apex of lateral margin rim with seven to ten rather long setae (in dorsal view). Clypeal suture rather feeble (discernible); anterior margin rim nearly transverse, but distinctly soared at the distal area of clypeal suture. Frontoclypeal suture nearly transverse, rather distinctly marked. Epistoma medial area weakly convex; clypeal surface distinctly microreticulated, rather densely punctate and its punctures denser and larger to laterally; Surface below the frontoclypeal suture with three protuberances, medial protuberance distinctly soared, lateral protuberances moderately soared. Frontal area with fine punctures, moderately to rather densely distributed; medial area surface smooth, without punctuation, and slightly pressed.

**Epipharynx** (Fig. 4C and D): Anterior margin slightly convex medially; lateral angles rounded; lateral margins moderately narrowed posteriorly. Epitorma-tylus structure rounded-triangular, slightly exceeding anterior margin of epipharynx; tylus area with two distinct lengths of celtes, closely grouped: one to two long celtes basally; three to five short celtes apically (median region). Epitorma anterolateral area with numerous heli. Acropariae with homogenous chaetae, moderately dense. Acanthopariae chaetae distinctly shorter than acropariae chaetae, becoming sparser posteriorly. Prophobae with microsetae moderately dense, denser proximally; chaetopedia moderately distributed in three to four irregular rows. Chaetopariae with dense row of stout spinules, slightly longer, but thinner than chaetopedia. Apophobae with a few rows of microsetae, moderately distributed along chaetopariae. Dexiotorma slightly longer than laeotorma.

**Pronotum** (Fig. 3A and B): Pronotum disc sub-rectangular (Fig. 3A), widest in posterior 1/3 area to posterior angle; pronotum maximum width very slightly narrower than that of the elytra. Pronotum margin rim distinct, moderately thick in lateral margin (Fig. 3B), very thin in posterior margin; anterior margin nearly transverse in dorsal view; anterior angle more weakly protruded than anterior margin; lateral margin very weakly arcuate; posterior angle widely round; posterior margin weakly sinuate, slightly convex at the middle, along the margin rim with very narrow and shallow furrow. Dorsal surface with two different-sized punctures; rather densely punctate, larger punctures 3x to 4x larger than fine punctures; punctures sparsely in medial area, denser to laterad.

**Scutellum** (Fig. 1A; Fig. 3A): Small and triangular; surface microreticulated; surface with few shallow irregular-shaped punctures distributed moderately.

**Elytra** (Fig. 3A and B): Moderately convex (Fig. 3A), very slightly widened posteriorly; without humeral teeth; with clearly visible ten striae intervals; most striae joined or shortened before apex. Striae very distinct and somewhat wide and deep; strial punctures very densely punctate, its moderately indenting margin of intervals at the anterior half; strial punctures distinctly larger than micropunctures of intervals. 1^st^ and 10^th^ striae mostly joined at the apical; remaining striae generally not joined, nearly confluent near the base (especially 9^th^ and 10^th^ striae); 4^th^ and 5^th^, 7^th^ to 9^th^ striae occasionally joined; [Second joining] (7^th^+8^th^) and 9^th^ striae occasionally joined. 6^th^ to 8^th^ striae shortened before basis, 6^th^ stria slightly longer than 7^th^ stria at the basis (Fig. 3B); 8^th^ stria starting from anterior 2/7 area of elytra. Intervals surface weakly microreticulated; central area somewhat distinctly convex (carinate), especially in posterior area, intervals lateral border rather flattened; micropunctures moderately punctate, forming three to five rows in intervals. Elytral margins border very adjacent with 10^th^ stria.

**Legs** (Fig. 1A; Fig. 3A and C): Meso- and metatrochanter with two to three rather long setae at the ventral ridges. Femora surface microreticulated and punctate finely and moderately distributed; profemoral ventral surface punctures larger than meso- and metafermoral ones, antero-ventral ridge with two rows of somewhat densely distributed long setae; ventral surface of mesofemora anterior area with three to five large setigerous punctures, medio-apical area with a row of two to three setigerous punctures and its setae long and stout (Fig. 3C). Protibiae (Fig. 3A) anterior area surface smooth; outer margin tridentate, remaining area with two to three fine teeth (smaller to proximally); apical spurs very slightly curved downwardly and apex relatively sharp, not reaching half of 2^nd^ protarsomere. Meso- and metatibiae with two strong transverse carinae; bristle of spinules rather long and two different lengths in carinae (occasionally subequal lengths); most apical rim spinules variable in length, usually two to three size classes, generally subeqaul; variation also within an individual, differences possibly due to abrasion, especially on the metatibial rim. Metatibiae superior apical spur as long as 1^st^ metatarsomere; inferior apical spur shorter than 1^st^ metatarsomere. 1^st^ metataromere (= basimetatarsomere) slightly longer than next two combined (Fig. 1A; Fig. 3A). Claws rather fine, regularly, arcuate.

**Venter** (Fig. 3C): Mesosternum with medial surface microreticulated; coarse, irregular punctures sparser medially, denser laterally; medial area with numerous very thin vertical furrows. Metasternum very shiny; medial area slightly elevated, surface weakly concave; with a thin vertical furrow medially; micropunctures moderately to rather densely distributed. Abdominal sternites weakly shiny, with distinct microreticulation; setigerous punctures rather densely distributed; setae of two distinct lengths, longer setae present on the medio-lateral areas of ventrites. Pygidium similar in structure to abdominal sternites; with six to eight long setae on the medial area; posterior margin with moderately dense, medium-length setae.

**Figure 5.**
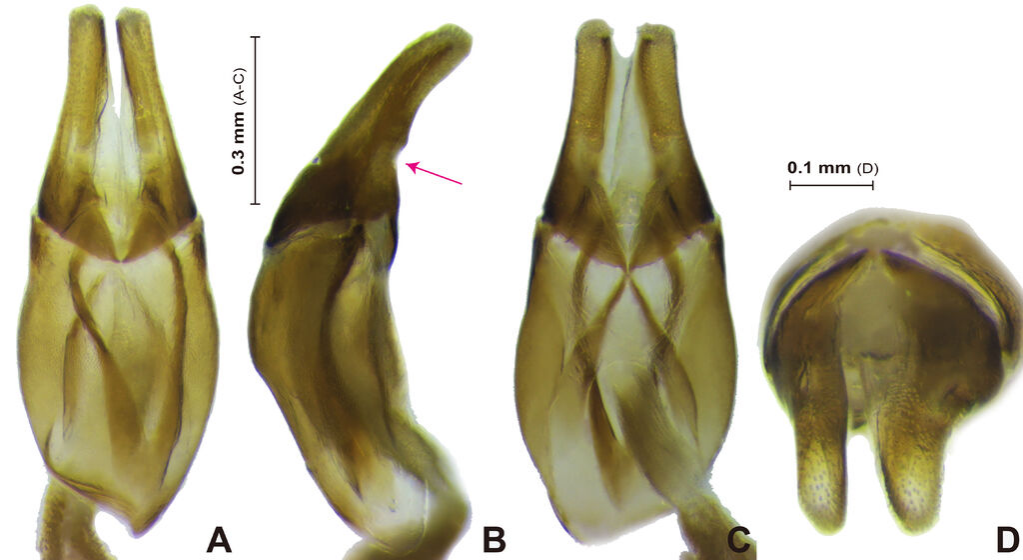
*Carinaulus inexpectatus*, male genitalia. **A** dorsal aspect; **B** lateral aspect; **C** ventral aspect; **D** apex of parameres. Red arrow = concave area.

**Aedeagus** (Fig. 5): Parameres symmetrical and simple shape (Fig. 5A); phallobase slightly longer than parameres length. Parameres slightly curved downwardly; gently narrowed anteriorly; ventral surface microgranulate (Fig. 5C); ventral area slightly concave at the basal 1/4 area (Fig. 5B); apex round and microgranulated (Fig. 5D).

**Female (sexual diamorphism)** (Fig. 2A, B; Fig. 4B). Similar to male, with the following differences.

**Head** (Fig. 4B): Frontoclypeal central protuberance less protruded than male. Head dorsal surface punctation slightly denser than male.

**Pronotum** (Fig. 2A, B): Pronotum dorsal punctation denser than male. Pronotum less convex than male.

**Venter**: Metasternum medial soared area, its surface nearly flattened.

#### Distribution

**Figure 6.**
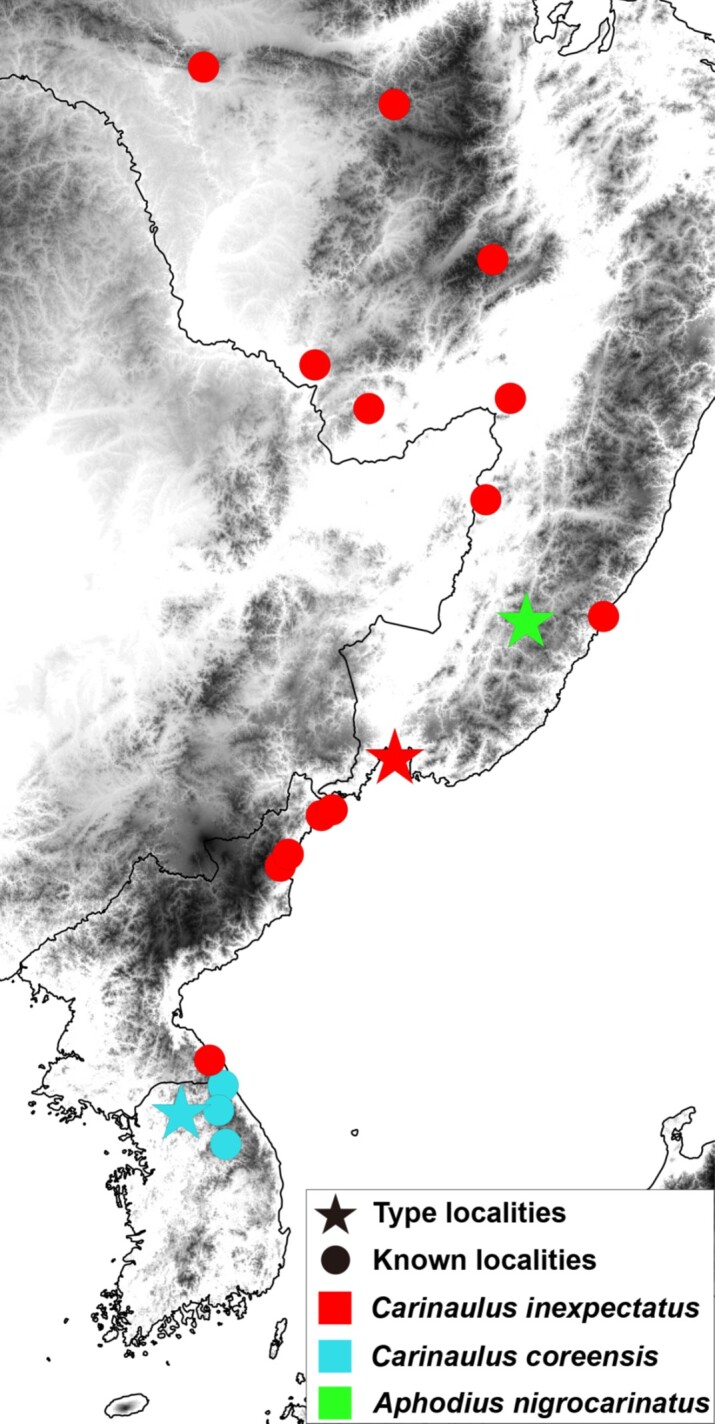
Distribution map of *Carinaulus inexpectatus* and its synonyms. Coloured stars indicate type localities of *C. inexpectatus* (red), *C. coreensis* (blue) and Aphodius (Carinaulus) nigrocarinatus (green), whereas coloured circles represent known localities of each taxon, based on examined specimens and literature records.

Korea (South, North), Russia (Far East) (Fig. 6).

#### Remarks

*Carinaulus coreensis* (Kim, 1986) was originally described, based on the holotype, allotype and 13 paratypes (Kim 1986). Examination of the designated type repository, the Sungshin Women’s University Insect Collection (SWUIC, Seoul), revealed that the holotype and majority of paratypes are not currently traceable. However, two female paratypes (Fig. 2) and one additional voucher specimen were recovered from the collection of the NIBR (Incheon). Additionally, one male and one female voucher specimen, identified by Kim, were located in the NASIC (Wanju). For comparative analyses, photographs of the holotype of *C. inexpectatus* (Balthasar, 1935), housed in the Czech National Museum (Prague), were examined (Fig. 1). Three additional male specimens collected from Mt. Geumgang, North Korea and housed in NIBR were also studied. These served as reference material for evaluating the taxonomic status of *C. coreensis*. See the discussion below for the justification of the synonymy.

## Discussion

A key diagnostic feature originally proposed by Kim (2012), that is, the relative length of apical spinules along the meso- and metatibial margins, was critically re-assessed. Kim (2012) diagnosed the two taxa as follows: apical spinules along the meso- and metatibial margins unequal in *C. coreensis* (consistent with the original description; Kim (1986)) and equal in *C. inexpectatus*. However, this character exhibited high variability, not only amongst individuals of *C. coreensis* (both sexes), but also within single specimens (left–right asymmetry and within-tibial variation) and in *C. inexpectatus* itself. The holotype of *C. inexpectatus* presented both short and long spinules, indicating that this trait is unreliable for distinguishing species. Comparative morphological analyses of all specimens revealed complete concordance in external characters, epipharyngeal morphology and male genitalia between *C. coreensis* and *C. inexpectatus*. These findings are consistent with previously published re-descriptions and illustrations (Stebnicka 1980, Stebnicka and Galante 1991, Minkina 2022). In the absence of stable diagnostic characters, we regard *Carinaulus coreensis* Kim, 1986 as a junior synonym of *Carinaulus inexpectatus* (Balthasar, 1935).

Historical inconsistencies in the recorded distribution of these species have contributed to longstanding taxonomic confusion. Dellacasa and Dellacasa (2006) listed *C. coreensis* as present in both North and South Korea [listed therein as Aphodius (C.) coreensis] and indicated that *C. inexpectatus* is limited to North Korea. In a later revision (Dellacasa et al. 2016), the *C. inexpectatus* distribution was expanded to both regions, while *C. coreensis* was limited to the south. The synonymy established here confirms that *C. inexpectatus* occurs throughout the Korean Peninsula. Importantly, many previous reports of distributions were not based on verifiable voucher specimens. Although recent catalogues and online databases (e.g. Dellacasa et al. (2016), Schoolmeesters (2025)) consistently list a broad distribution encompassing Japan, East Siberia and Kazakhstan, no primary evidence supports these records. In Stebnicka and Galante (1991), the reference to “south-eastern Siberia” clearly denotes Primorsky Kray (Amur–Ussuri lands) and Honshu, Japan was mentioned in Stebnicka (1980) without any specimen evidence. Moreover, the citation of Japan in Stebnicka and Galante (1991) likely resulted from a miscitation or misinterpretation of Stebnicka (1980), as Honshu is not listed in the distribution table or elsewhere in the original work (see Stebnicka (1980): 206, Table 2). Consequently, the Japanese and Siberian records are considered erroneous and the occurrence in Kazakhstan remains unverified pending examination of primary specimens. The confirmed distribution of *C. inexpectatus* is, therefore, restricted to the Korean Peninsula and the Russian Far East (Bezborodov and Berlov 2005, Bezborodov 2012, Akhmetova and Frolov 2014, Bezborodov 2014, Shabalin 2022, Fig. 6).

## Supplementary Material

XML Treatment for Carinaulus

XML Treatment for Carinaulus inexpectatus
